# Microsurgical clipping as a retreatment strategy for previously ruptured aneurysms treated with the Woven EndoBridge (WEB) device: a mono-institutional case series

**DOI:** 10.1007/s00701-023-05596-5

**Published:** 2023-05-13

**Authors:** B. Kranawetter, S. Hernández, D. Mielke, M.S. Ernst, V. Malinova, V. Rohde

**Affiliations:** 1 Department of Neurosurgery, University Medical Center, Georg-August University Göttingen, Göttingen, Germany; 2 Department of Neuroradiology, University Medical Center, Georg-August University Göttingen, Göttingen, Germany

**Keywords:** Woven EndoBridge, Subarachnoid hemorrhage, Ruptured aneurysm, Microsurgical clipping, Recurrent aneurysm, Residual aneurysm, Retreatment of aneurysms

## Abstract

**Background:**

Since its approval by the US Food and Drug Administration (FDA) in 2018, the flow disruptor Woven EndoBridge (WEB) device has become increasingly popular for the endovascular treatment of unruptured and ruptured cerebral aneurysms. However, the occlusion rates seem rather low and the retreatment rates rather high compared to other treatment methods. For initially ruptured aneurysms, a retreatment rate of 13 % has been reported. A variety of retreatment strategies has been proposed; however, there is a paucity of data concerning microsurgical clipping of WEB-pretreated aneurysms, especially previously ruptured ones. Thus, we present a single-center series of five ruptured aneurysms treated with the WEB device and retreated with microsurgical clipping.

**Methods:**

A retrospective study including all patients presenting with a ruptured aneurysm undergoing WEB treatment at our institution between 2019 and 2021 was performed. Subsequently, all patients with an aneurysm remnant or recurrence of the target aneurysm retreated with microsurgical clipping were identified.

**Results:**

Overall, five patients with a ruptured aneurysm treated with WEB and retreated with microsurgical clipping were included. Besides one basilar apex aneurysm, all aneurysms were located at the anterior communicating artery (AComA) complex. All aneurysms were wide-necked with a mean dome-to-neck ratio of 1.5. Clipping was feasible and safe in all aneurysms, and complete occlusion was achieved in 4 of 5 aneurysms.

**Conclusions:**

Microsurgical clipping for initially ruptured WEB-treated aneurysms is a feasible, safe, and effective treatment method in well-selected patients.

## Introduction

Intrasaccular flow disruption describes an endovascular treatment method, where in contrast to flow diversion, the device is directly placed into the aneurysm sac [28]. In 2018, the flow disruptor Woven EndoBridge (WEB; MicroVention, Aliso Viejo, California, USA) has been approved as the first intrasaccular device for the treatment of unruptured wide-necked bifurcation aneurysms (WNBAs) [28] by the FDA. Due to deployment within the aneurysm sac, anticoagulation can be limited to periprocedural heparinization [37] which makes the WEB device also suitable for the treatment of ruptured aneurysms. Thus, endovascular centers have used WEB off-label in the setting of subarachnoid hemorrhage (SAH), and several retrospective studies have demonstrated a good safety and efficacy of the device [, , 12, 15, 38]. More recently, the data from the prospective “CLinical Assessment of WEB device in Ruptured aneurYSms” (CLARYS) study have been published [34]. There was a complete occlusion rate of 41.3%; however, it seems rather low, especially when compared to the well-known occlusion rates of microsurgical clipping. In the 6-year follow-up of the Barrow Ruptured Aneurysm Trial (BRAT), for example, a complete occlusion rate of 96 % after microsurgical clipping has been demonstrated [33]. Furthermore, a retreatment rate of 13 % within the first year seems rather high compared to 3.8 % of patients undergoing retreatment in BRAT [, 5, 34]. In the CLARYS study, retreatments were performed by simple coiling in one case, stent-assisted coiling in four cases, and by flow diversion in one case [34]. None of the aneurysms was retreated with microsurgical clipping. In general, there is a paucity of data concerning microsurgical clipping of WEB-pretreated aneurysms, especially previously ruptured ones. In three recent, large multicenter studies, 0.8 % (6/756) [6], 1.4% (10/691) [14], and 2% (6/342) [36] of WEB-treated aneurysms were retreated with microsurgical clipping. Previous rupture of the aneurysms retreated with microsurgical clipping has been mentioned in two of the studies and was 0.6% (4/691) [14] and 0.9% (3/342) [36], respectively. However, detailed data on the technical aspects of microsurgical clipping and outcome have been lacking [, 14, 36]. Therefore, we present a mono-institutional case series of five ruptured WEB-treated aneurysms that have been retreated with microsurgical clipping. The aim of the study was to demonstrate the intraoperative findings, the feasibility, the efficacy, and the safety of clipping after primary WEB treatment of ruptured aneurysms.

## Materials and methods

### Patient population

A retrospective study of consecutive adult patients who presented with a ruptured aneurysm and underwent WEB treatment at our institution between 2019 and 2021 was performed. Subsequently, we identified all patients who had a recurrence or a residual of the target aneurysm and underwent retreatment with microsurgical clipping. Of these patients, demographic data, the World Federation of Neurosurgical Societies (WFNS) grade status at admission, Fisher grade on initial computed tomography (CT) scan, aneurysm morphology prior to initial aneurysm treatment (size, neck size, dome-to-neck ratio), data of initial endovascular treatment (type and size of WEB, procedure-related complications), surgical data (approach, technical aspects, operation-associated complications), and outcome using the modified Rankin Scale (mRS) were documented. The study complied with the Declaration of Helsinki and was approved by the local ethics review committee. Patient’s consent for treatment was obtained according to the individual institutional guidelines. Due to the retrospective analysis of the data for this study, additional informed consent was deemed unnecessary. All patients were treated according to our institutional treatment protocol which has been described before [, 20, 21] and aligns with current guidelines for the management of aneurysmal SAH [10].

### Treatment strategy

Every aneurysm case was discussed in a neurovascular board consisting of a senior neurosurgeon, neuroradiologist, and neurologist. Endovascular treatment only was considered as a valid treatment option if no peri- and postprocedural dual antiplatelet therapy (DAPT) would be required. The final decision for WEB over coil has been at the discretion of the neurointerventionalist. Device selection and standard WEB device deployment has been used in all cases [28]. WEB treatment was performed under general anesthesia, and all procedures were done by femoral artery access. Periprocedural anticoagulation was limited to heparin. In all patients, a follow-up digital subtraction angiography (DSA) was performed 6 months after WEB treatment. The status of occlusion was evaluated using the Raymond–Roy occlusion classification (RROC) [22] and WEB occlusion scale (WOS) [4]. If an aneurysm remnant or recurrence was demonstrated, the case was re-discussed in the neurovascular board where the decision for retreatment and retreatment strategy was made. In all patients, coiling without additional devices was seen as not feasible due to the wide-neck configuration of the included aneurysms. Stent-assisted coiling or a flow-diverter stent were considered to be associated with a high treatment risk compared to surgery. Therefore, the consensus was reached to treat the re-perfused aneurysms by microsurgery.

## Results

### Case 1

**Fig. 1:**
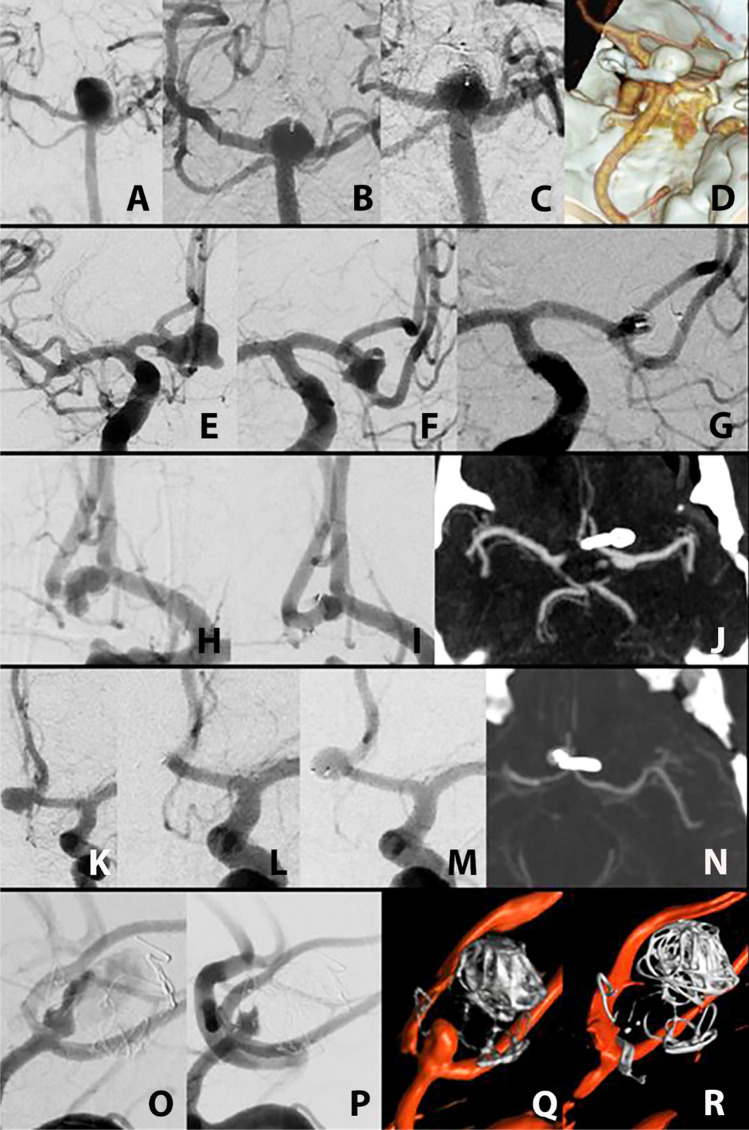
Course from the initial angiogram to the final outcome in every patient. **A**–**D** (patient 1): **A** shows the untreated basilar tip aneurysm, **B** a neck remnant equivalent to RROC II or WOS C after WEB, **C** the deformation of the device and progression to WOS D, and **D** the result after clipping. **E**–**G** (patient 2): **E** shows the AcomA aneurysm on initial DSA, **F** the radiopaque marker of the WEB protruding into the neck, and **G** demonstrates the position of the clip and the small neck remnant. **H**–**-J** (patient 3): **H** demonstrates the AComA aneurysm prior to treatment, **I** the neck remnant after WEB, and **J** the clip placement on CTA. **K**–**N** (patient 4): **K** demonstrates the untreated AcomA aneurysm, **L** complete occlusion after WEB, **M** the deformation of the device with complete refilling of the aneurysm, and **N** the result after clipping. **O**–**R** (patient 5): **O** demonstrates the reperfusion of the large initially ruptured and coiled AComA aneurysm, **P** the result after placement of the WEB within the aneurysm neck, **Q** the reperfusion of the aneurysm neck, and **R** the final result after clipping on 3D angiography

The 48-year-old female patient presented with aneurysmal SAH WFNS grade II, Fisher grade 3 caused by a ruptured basilar tip aneurysm with a maximal diameter of 9 mm, a neck diameter of 8 mm, and a dome-to-neck ratio of 1.3 (Fig. 1). Besides the basilar apex aneurysm, the patient was diagnosed with two other aneurysms located at the internal carotid artery (ICA) on each side. The basilar apex aneurysm was uneventfully treated with a WEB SL 9 × 5 mm; during the procedure, heparin was given. The final angiogram demonstrated residual flow within the device. No procedure-related complications occurred. The first follow-up DSA 9 months later already demonstrated residual filling of the aneurysm neck equivalent to RROC II or WOS C (Fig. 1). After interdisciplinary case discussion, aneurysm retreatment was postponed waiting for full thrombosis of the aneurysm. However, the second DSA showed a progression to WOS D with a compaction and deformation of the WEB device (Fig. 1) ,and microsurgical retreatment was planned.

### Case 2

The 61-year-old female patient had an aneurysmal WFNS grade I and Fisher grade 1 SAH, proven by lumbar puncture. DSA demonstrated a ruptured AComA aneurysm with a maximal diameter of 14 mm, a neck diameter of 8 mm, and a dome-to-neck ratio of 1.8 (Fig. 1). The aneurysm was treated with a WEB SL 10 × 5 mm. At first, a WEB SL 10 × 8 mm was positioned within the aneurysm sac but did not expand appropriately so that the smaller 10 × 5 mm WEB was used as the final device; during the procedure, heparin was given. On the final angiogram, residual filling was shown, however, with stasis within the device. No procedure-related complications occurred. Follow-up DSA 7 months later demonstrated a neck remnant distal to the radiopaque marker equivalent to RROC II and WOS C (Fig. 1) without a chance of delayed complete aneurysm occlusion. Therefore, microsurgical retreatment was planned.

### Case 3

The 62-year-old male patient presented with aneurysmal WFNS grade I and Fisher grade 3 SAH. CTA and DSA revealed a ruptured AComA aneurysm with a maximal diameter of 5mm, a neck diameter of 3 mm, and dome-to-neck ratio of 1.3 as the source of the bleeding (Fig. 1). The patient was uneventfully treated with a WEB SL 5 × 3 mm; during the procedure, heparin was given. Follow-up DSA 9 months after WEB demonstrated a reperfusion of the aneurysm RROC II or WOS C (Fig. 1). The decision for microsurgical retreatment was made.

### Case 4

The 54-year-old female patient was admitted with a WFNS grade I and Fisher grade 2 SAH, caused by a ruptured AComA aneurysm with a maximal diameter of 9 mm, a neck diameter of 4 mm, and a dome-to-neck ratio of 1.6 (Fig. 1). The aneurysm was uneventfully treated with a WEB SL 7 × 4 mm; during the procedure, heparin was given. The final angiogram showed complete occlusion of the aneurysm. DSA 8 months after endovascular treatment demonstrated a compaction and deformation of the WEB with complete refilling of the aneurysm equivalent to RROC III or WOS D (Fig. 1). The decision for microsurgical retreatment was made.

### Case 5

The 63-year-old female patient was diagnosed with a WFNS grade V and Fisher grade 4 SAH caused by a ruptured AComA aneurysm with a maximal diameter of 9mm, a neck diameter of 5, and a dome-to-neck ratio of 1.8. The initial treatment consisted of coiling. A neck remnant then was treated with a WEB SL 5 × 3mm; during the procedure, heparin was given. The final angiogram showed complete occlusion of the aneurysm. No procedure-related complications occurred. Follow-up angiography was done 6 months later; however, it demonstrated a residual filling of the aneurysm neck equivalent to RROC II or WOS C (Fig. 1). Microsurgical retreatment was planned.

### Microsurgical clipping, intraoperative findings, and outcome

**Fig. 2:**
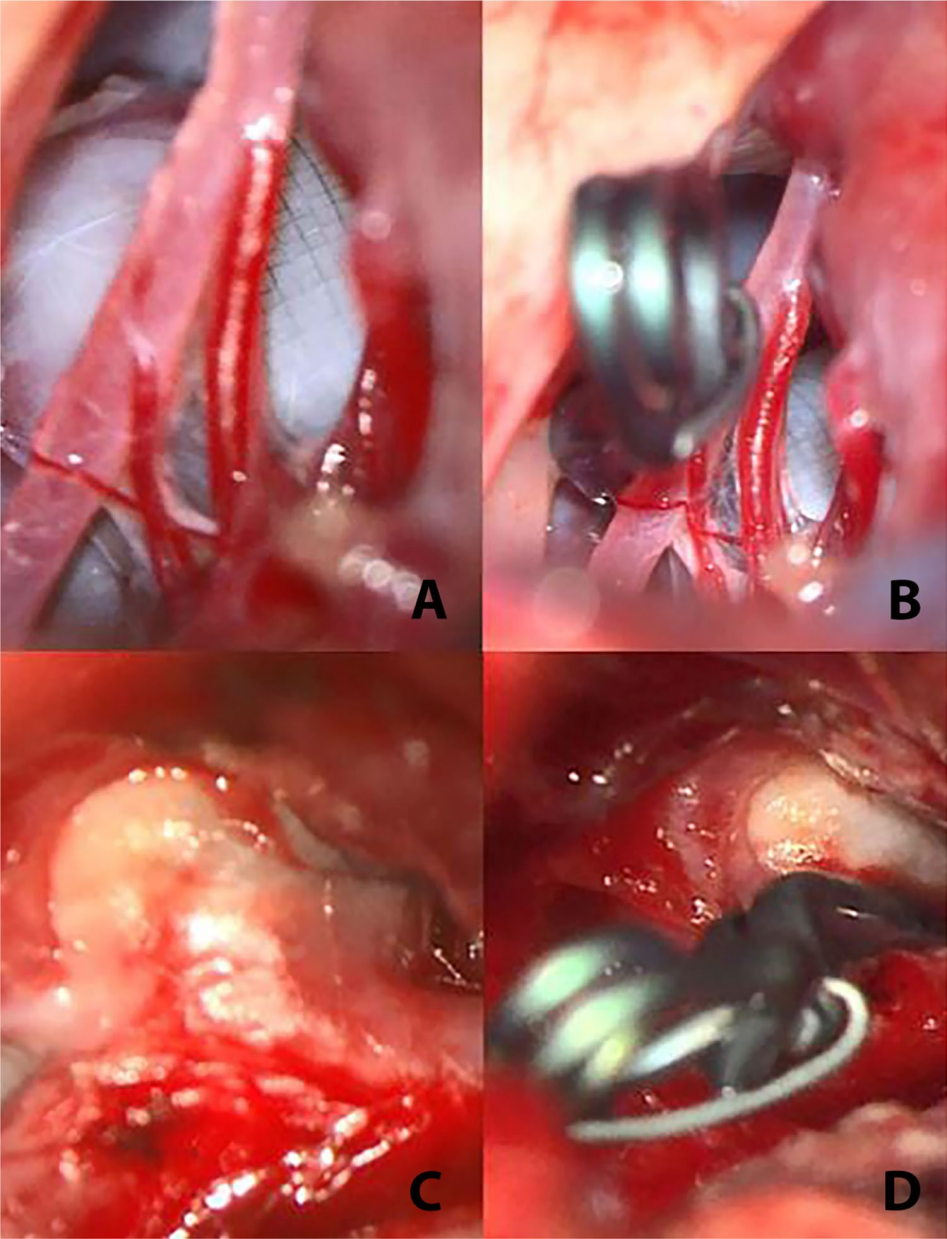
**A**–**D** shows the intraoperative findings in two patients before and after clip placement. The mash of the WEB shines through the aneurysm wall in both patients

For the clipping of the AComA aneurysms, a standard pterional approach was used. For the basilar apex aneurysm, a pterional approach with temporal extension was performed. Dissection and visualization of the aneurysm was feasible in all cases but was challenging in patient 2 due to severe adhesions in the region of the aneurysm dome. In all cases, the braided mesh of the WEB device was visible through the thin aneurysmal wall but did not penetrate into the subarachnoid space (Fig. 2). Additionally, residual filling of the aneurysm base with pulsation was visible in all patients and was verified by indocyanine green (ICG) angiography prior to clipping. Manipulation or removal of the device was not necessary in any case. After direct visualization of the aneurysm neck, occlusion of the aneurysm with one titanium clip was possible. Micro-Doppler sonography demonstrated both parent and distal vessel patency, and ICG angiography showed complete aneurysm occlusion in four patients. In patient 2, ICG demonstrated complete occlusion of the aneurysm dome. However, the aneurysm clip could not be positioned as intended across the neck due to the radiopaque marker of the WEB device, and a small, not accessible aneurysm remnant was assumed. CTA demonstrated complete aneurysm occlusion in all cases. Due to the intraoperative impression of an aneurysm remnant, postoperative DSA was additionally performed in patient 2, confirming the small neck remnant and the technical difficulty of clipping the aneurysm completely due to the radiopaque marker of the WEB protruding downwards to the level of the neck. Overall, three procedure-related complications occurred. There were two surgical site infections (SSI), one was successfully treated with antibiotics (patient 2), but one patient (patient 1) required surgical wound revision. The third complication was severe vasospasm. On day 3 after clipping, the patient (patient 5) experienced hypotension, which was associated with aphasia and a right sided hemiparesis. A CCT/CTA and CT perfusion demonstrated delayed perfusion within the left-sided anterior cerebral artery (ACA) territory and severe vasospasm of the left A2-segment. Thus, invasive vasospasmolysis with intra-arterial nimodipine and induced hypertension were performed, leading to complete recovery within 3 days. Two patients (patient 3, patient 5) were left with a mild cognitive impairment equivalent to a modified Rankin scale (mRS) 1 due to the SAH. The other three patients recovered completely (mRS 0).

## Discussion

Since its FDA approval for the treatment of unruptured WNBA in 2018, the WEB device has gained increasing popularity for the endovascular treatment of aneurysms in Europe as well as in the USA [28]. Its safety and efficacy for the treatment of unruptured aneurysms have been evaluated in several prospective multicenter studies [, 4, 30]. Due to the relatively fast and easy device deployment and the lacking necessity of antiplatelet therapy, the WEB has also become widely used in the setting of aneurysmal SAH [15]. However, by now, only one prospective study (CLARYS) has evaluated the WEB in the treatment of ruptured aneurysms with a limited follow-up period of 12 months [34]. So far, the reported complete occlusion rate seems to be rather low compared to other treatment methods, especially microsurgical clipping [, , 18, 23, 33]. Although some interventionalists have postulated that neck and aneurysm remnants after WEB treatment may occlude over a longer time period, current available data, however, rather indicates the opposite: at 3 years, complete occlusion in 50.8%, neck remnant in 32.8 %, and aneurysm remnant in 16.4 % have been reported in the cumulative population of the WEBCAST and WEBCAST 2 study [31]. After 5 years, complete aneurysm occlusion was seen in 51.6%, neck remnants in 26.3%, and an aneurysm remnant in 22.1% [32]. The retreatment rate was 11.4 % and 11.6% after 3 and 5 years of follow-up, respectively [, 31, 32]. The latest results from a French multicenter database including 756 WEB-treated aneurysms have shown a retreatment rate of 7.5 % [6]. A recently published US multicenter study, including 342 aneurysm, demonstrates similar results with a retreatment rate of 9 % (30/342) [36]. A higher retreatment risk for ruptured than for unruptured aneurysms has been demonstrated in both studies [, 6, 36], and retreatment rates have been similar to the 13 % (6/46) reported in the prospective CLARYS study [34]. In contrast to retreatment rates of <1% and 3.8% in large, prospective studies evaluating microsurgical clipping of ruptured aneurysms, the retreatment rate for WEB-treated ruptured aneurysms seems relatively high [, 23, 35].

### Criteria for complete occlusion and indication for retreatment

Over the past decades, the RROC [22] has become the gold standard for the definition of aneurysm occlusion after endovascular treatment with coiling. However, in prior studies using WEB, such as the WEB-IT study, the angiographic outcome was evaluated using the WOS [4]. The WOS, however, modifies the RROC and considers complete occlusion as WOS A and B, where WOS B describes opacification of the proximal recess of the device. “Adequate” occlusion additionally includes WOS C which describes a neck remnant equivalent to RROC II [4]. This is a very controversial topic and has already been widely discussed within the scientific community since it has caused confusion regarding the definition of a neck remnant. The term “adequate” is supposed to define a stable aneurysm without the necessity for retreatment. However, other series [36] as well as ours have demonstrated that aneurysms with a WOS C may progress to a higher grade over time and will need retreatment in the future, which implies that “adequate” occlusion might not be adequate after all and that neck remnants from WEB should be followed up closely. Whereas the risk of re-rupture from neck remnants after coiling and the retreatment indications after coiling for primary ruptured aneurysms have been well-established, this still needs to be determined for the WEB [25]. However, what previous studies have already demonstrated is that, similar to endovascular coiling, retreatment rates are higher in initially ruptured aneurysms treated with WEB [, , 6, 25, 36].

### Retreatment strategies

A variety of retreatment modalities after WEB including coiling, stent-assisted coiling, flow-diverter stents, a second WEB, and microsurgical clipping have been proposed [, 6, 36]. Retreatment, however, depends on several factors such as location of the aneurysm, size, and feasibility of surgery. Srinivasan et al. [36] proposed a simplified retreatment algorithm after initial WEB treatment. According to their algorithm, clipping should be chosen as the first treatment option for all eligible candidates since it the most definitive treatment method. However, even in their own study, the majority of aneurysms have been retreated with endovascular strategies, and only a small percentage was retreated with microsurgical clipping, mostly due to a broad-necked configuration and the location of the aneurysm [36]. In general, the expertise regarding microsurgical clipping as a retreatment method for previously ruptured aneurysms treated with WEB is limited. Although larger multicenter studies have mentioned the clipping of initially ruptured WEB-treated aneurysms as demonstrated in Table 1, there have been lacking a detailed description of the intraoperative course, surgical technique, and outcome [, , 14, 19, 36]. Thus, to our best knowledge, this study currently represents the largest single-institution, single-surgeon series of ruptured aneurysms initially treated with WEB and retreated with microsurgical clipping.

**Table 1:** Summary of the recent literature evaluating the WEB device for the treatment of unruptured and ruptured aneurysms. This further demonstrates that microsurgical clipping as a retreatment strategy for ruptured WEB-pretreated aneurysms has only been used in a few cases or has not been described at all in most studies

Author	Aneurysms* *n*=	Ruptured aneurysms *n*=(%)	Retreated aneurysms *n*=(%)	Ruptured aneurysms retreated with clipping *n*=(%)	Location *n*=(%)		
					AComA	MCA	BA
Kortman et al., 2022 [19]	78	78 (100)	5 (6)	1 (1.2)	n/a	n/a	n/a
Alpay et al., 2022 [3]	66	25 (38)	8 (12)	n/a	n/a	n/a	n/a
Caroff et al., 2022 [6]	756	294 (39)	57 (8)	n/a	n/a	n/a	n/a
Alpay et al., 2022 [2]	91	28 (31)	13 (14)	n/a	n/a	n/a	n/a
Spelle et al., 2022 [34]	60	60 (100)	6 (10)	n/a	n/a	n/a	n/a
Diestro et al., 2022 [14]	691	162 (23)	51 (7)	4 (0.6)	n/a	n/a	n/a
Pierot et al., 2022 [32]	95	7 (7)	11 (12)	n/a	n/a	n/a	n/a
Goertz et al., 2022 [16]	94	54 (57)	11 (12)	n/a	n/a	n/a	n/a
Cortese et al., 2022 [11]	215	59 (27)	20 (9)	n/a	n/a	n/a	n/a
Kewlani et al., 2022 [17]	74	28 (37)	6(8)	n/a	n/a	n/a	n/a
Srinivasan et al., 2022 [36]	342	13 (4)	30 (9)	3 (0.9)	2 (0.5)	1 (0.2)	n/a
Algin et al., 2022 [1]	83	49 (59)	4 (5)	n/a	n/a	n/a	n/a
Mouchtouris et al., 2021 [24]	115	34 (30)	6 (5)	n/a	n/a	n/a	n/a
Pagano et al., 2021 [27]	92	6 (7)	2 (2)	n/a	n/a	n/a	n/a
Cherian et al., 2021 [9]	91	8 (9)	4 (4)	n/a	n/a	n/a	n/a
Cortez et al., 2021 [12]	94	94 (100)	4 (4)	n/a	n/a	n/a	n/a
Youssef et al., 2021 [39]	48	48 (100)	2 (4)	n/a	n/a	n/a	n/a
Pierot et al., 2020 [30]	152	8 (5)	14 (9)	n/a	n/a	n/a	n/a
Arthur et al., 2019 [4]	143	8 (6)	14 (10)	n/a	n/a	n/a	n/a
Pierot et al., 2019 [29]	4	1 (25)	4 (100)	1 (25)	1 (25)	n/a	n/a
Kranawetter et al., 2023*	5	5 (100)	5 (100)	5 (100)	4 (80)	n/a	1 (20)

*Aneurysms with evaluation of retreatment

*The presented series

Previous aneurysm rupture may be of significant interest to the surgeon since it may increase the complexity and the risk of surgery [26]. The clipping of primary coiled aneurysms has been described as technically challenging in unruptured and even riskier or not feasible in initially ruptured coiled aneurysms [26]. Different factors including compacted coils present in the aneurysm sac, slipping of the clip blades, transmural calcification, scarring, or coil loops within the aneurysm neck may complicate clip application [13]. Prior SAH may further compound these challenges due to increased scaring and adhesions, as we have witnessed in one patient [26]. Our intraoperative findings confirmed that WEB becomes integrated into the aneurysm wall and that it does not penetrate into the subarachnoid space in ruptured aneurysms as it has been described before [29]. We showed that safe clipping without any complications is feasible. However, when surgery is selected as a retreatment strategy, the position of the radiopaque marker within the aneurysm should be considered: in one of our cases, the radiopaque marker, which was protruding down to the neck level, hindered optimal clip placement and led to a small aneurysm remnant. According to our experience, manipulation of the device or even its removal seems not to be necessary for successful clipping. This might be of significance since the removal of coils for clip placement has been reported to be associated with a high morbidity [13]. Another reported issue with primary coiled aneurysms is that they can become stiff and resistant to manipulation which can make it challenging to access the aneurysm neck in narrow anatomical corridors. This may further lead to an increased risk of intraoperative rupture [8]. However, this does not seem to pose a problem with the WEB device. In conclusion, the surgical morbidity of microsurgical treatment of ruptured aneurysms pretreated with a WEB may be smaller than that of previously coiled aneurysms. Overall, our results from this case series suggest that microsurgical clipping after WEB treatment in initially ruptured aneurysms is well executable with good clinical results and high occlusion rates. However, the good results should not be mistaken as a parameter for a less complex operation.

### Institutional decision-making process

Our own institutional experience has shown that a significant proportion of WEB-treated aneurysms are left with a neck remnant or a residual aneurysm in the routinely performed angiography 6 months after the initial treatment. Thus, we did evaluate our own results after microsurgical clipping of incidental WNBAs and compared them with the outcome after WEB treatment in the 3 European landmark studies [7]. The morbidity has been low with both methods, but the complete occlusion rate has been significantly higher after microsurgical clipping (99% vs. 58%). Therefore, we became more restrained regarding the indication for a WEB device at our institution. Today, we preferably proceed to surgery if simple coiling cannot treat a WNBA, with the only exception being a wide-necked basilar tip aneurysm.

## Conclusion

Due to the increasing use of the WEB device, retreatment and choosing a retreatment strategy will become more frequent in the future. Our case series demonstrates that microsurgical clipping of initially ruptured WEB-treated aneurysms is a feasible, safe, and effective treatment method and that it is a reasonable alternative to endovascular retreatment strategies in well-selected patients.

### Limitations

One limitation of this study is that it only represents results in five patients. Further, our series may be biased by the fact that all aneurysms were treated by one senior neurosurgeon with expertise in vascular neurosurgery and that our results may not be replicable in other vascular centers. We also acknowledge the fact that postoperative angiography instead of postoperative CTA would have been the more vigorous imaging modality for proofing complete aneurysm occlusion. However, as per institutional standard operative procedure, angiography is only performed, if the surgeon remains in doubt of complete aneurysm occlusion.

## Abbreviations

ACA: Anterior cerebral artery
AComA: Anterior communicating artery
BRAT: Barrow Ruptured Aneurysm Trial
CT: Computed tomography
CTA: Computed tomography angiography
CLARYS: CLinical Assessment of WEB device in Ruptured aneurYSms
DSA: Digital subtraction angiography
DAPT: Dual antiplatelet therapy
EVD: External ventricular drain
FDA: Food and Drug Administration
GCP: Good clinical practice
ICG: Indocyanine green
ICA: Internal carotid artery
ICU: Intensive care unit
ICP: Intracranial pressure
MCA: Middle cerebral artery
MRA: Magnetic resonance angiography
MRI: Magnetic resonance imaging
mRS: Modified Rankin scale
RROC: Raymond–Roy Occlusion Classification
SAH: Subarachnoid hemorrhage
SSI: Surgical site infections
TCD: Transcranial Doppler
WOS: WEB occlusion scale
WFNS: World Federation of Neurosurgical Societies
WNBA: Wide-necked bifurcation aneurysm
WEB: Woven EndoBridge
